# Transcriptional activity of repair, apoptosis and cell cycle genes (TP53, MDM2, ATM, BAX, BCL-2, CDKN1A, OGG1, XPC, PADI4, MAPK8, NF-KB1, STAT3, GATA3) in chronically exposed persons with different intensity of apoptosis of peripheral blood lymphocytes

**DOI:** 10.18699/VJGB-22-08

**Published:** 2022-02

**Authors:** V.S. Nikiforov, E.A. Blinova, A.I. Kotikova, A.V. Akleyev

**Affiliations:** Urals Research Center for Radiation Medicine, Chelyabinsk, Russia; Chelyabinsk State University, Chelyabinsk, Russia; Urals Research Center for Radiation Medicine, Chelyabinsk, Russia; Chelyabinsk State University, Chelyabinsk, Russia; Urals Research Center for Radiation Medicine, Chelyabinsk, Russia; Chelyabinsk State University, Chelyabinsk, Russia; Urals Research Center for Radiation Medicine, Chelyabinsk, Russia; Chelyabinsk State University, Chelyabinsk, Russia

**Keywords:** mRNA, apoptosis, necrosis, lymphocytes, chronic exposure, мРНК, апоптоз, некроз, лимфоциты, хроническое облучение

## Abstract

Transcriptional activity of genes involved in maintaining genetic homeostasis (genes for repair, cell cycle and apoptosis: TP53, MDM2, ATM, BAX, BCL-2, CDKN1A, OGG1, XPC, PADI4, MAPK8, NF-KB1, STAT3, GATA3) was studied in chronically exposed persons with an increased intensity of early and late stages of apoptosis and necrosis of peripheral blood lymphocytes. The object of this study was peripheral blood mononuclear cells obtained from 132 chronically exposed residents of the Techa riverside villages. The mean accumulated dose to red bone marrow was 426.4 ± 48.2 mGy (1.3–2930.0 mGy), to thymus and peripheral immune organs, 58.9 ± 7.9 mGy (0.1–489.0 mGy). The study was performed more than 60 years after the onset of exposure, the average age of exposed persons was 68 ± 0.6 years (55–86 years). The study of apoptotic and necrotic death of peripheral blood lymphocytes was based on the presence of phosphatidylserine on the cell membrane surface, as well as on its permeability for DNA-intercalating dye. Evaluation of the relative content of mRNA genes for repair, cell cycle, and apoptosis was carried out using real-time PCR. An increased relative content of PADI4 gene mRNA was registered in the group of chronically exposed persons with the increased intensity of early apoptosis (p = 0.006). Modulation of the relative content of mRNA of the TP53 (p = 0.013) and BCL-2 (p = 0.021) genes was detected in the group of chronically exposed individuals with the increased intensity of the late stage of apoptosis. A statistically signif icant increase in the transcriptional activity of the TP53 gene was observed in the group of chronically exposed persons with the increased intensity of peripheral blood lymphocyte necrosis in the long-term period (p = 0.015). In the course of the study it was noted that exposed people with increased intensity of apoptosis, f irst of all, demonstrate changes in the transcriptional activity of apoptotic genes. These data are consistent with current views on the activation of programmed cell death.

## Abstract (ru)

Исследовали транскрипционную активность генов, вовлеченных в поддержание генетического гомеостаза клетки (репарации, клеточного цикла и апоптоза: TP53, MDM2, ATM, BAX, BCL-2, CDKN1A, OGG1, XPC, PADI4, MAPK8, NF-KB1, STAT3, GATA3), у лиц, подвергшихся хроническому радиационному облучению, с повышенной интенсивностью раннего, позднего апоптоза и некроза лимфоцитов периферической крови. Объектом изучения служили мононуклеарные клетки периферической крови, полученные от 132 жителей прибрежных сел реки Течи, подвергшихся хроническому облучению. Доза облучения красного костного мозга составляла 426.4 ± 48.2 мГр (1.3–2930.0 мГр), доза облучения тимуса и периферических органов иммунной системы – 58.9 ± 7.9 мГр (0.1–489.0 мГр). Исследование проводили в отдаленные сроки (более 60 лет с начала облучения), возраст людей на время проведения обследования был 68 ± 0.6 года (55–86 лет). Анализ апопто- тической и некротической гибели лимфоцитов периферической крови основывался на наличии на поверхности мембраны клеток фосфолипида фосфатидилсерина, а также ее проницаемости для интеркалирующего ДНК-красителя. Оценку относительного содержания мРНК генов репарации, клеточного цикла и апоптоза проводили с использованием полимеразной цепной реакции в реальном времени. В группе хронически облученных людей с повышенной интенсивностью раннего апоптоза отмечено увеличение относительного содержания мРНК гена PADI4 (p = 0.006). Для хронически облученных людей с повышенной интенсивностью позднего апоптоза зафиксирована модуляция относительного содержания мРНК генов TP53 (p = 0.013) и BCL- 2 (p = 0.021). В отдаленные сроки у хронически облученных людей с повышенной интенсивностью некроза лимфоцитов периферической крови отмечен статистически значимый рост транскрипционной активности гена TP53 (p = 0.015). Установлено, что у облученных людей с повышенной интенсивностью апоптоза регистрируются в первую очередь изменения со стороны транскрипционной активности апоптотических генов, что согласуется с существующими представлениями об активации программированной гибели клеток.

## Introduction

Ionizing radiation is the factor that could trigger changes in transcriptional activity of genes that have a key role in maintaining the stability of cellular homeostasis (Kabacik et al., 2011). Complex molecular responses to genotoxic stress set into action a lot of regulatory mechanisms including apoptosis (Zeegers et al., 2017).

Apoptosis plays an important part in the development of both early and late effects of ionizing radiation (Verheij, Bartelink, 2000). Its activation starts with changes in the expression of the genes regulating the processes of DNA damage reparation, cell cycle control, cell proliferation and differentiation, etc (Verheij, Bartelink, 2000). With cell death, a genetic program regulating the balance of intracellular pro- and anti-apoptosis factors starts developing. At the early stage of apoptosis, the expression of phosphatidylserine begins on the external surface of the membrane. However, its presence is not the strict requirement of cell death. Of great importance are its concentration and formation of a complex with other proteins. It sends a signal to the phagocytes to recognize the apoptotic cells (Bevers, Williamson, 2016).

Protein p53 that regulates apoptotic genes, coding cells of cellular membrane (CD95, DR5), proteins of cytoplasm and proteins located on the metachondrial membrane (proteins of the BCL-2 family), plays an important role in the activation of signaling cascade that induces apoptotic cell death (Chipuk, 2006). Moreover, the BAX/BCL-2 protein ratio predetermines the implementation of apoptotic cell death. It was demonstrated that in case of ionizing radiation apoptosis is initiated against the early repression of the BAX gene and increase in the activity of BCL-2 in human blood cells (Azimian et al., 2015).

In physiological conditions, a strict balance of pro- and antiapoptotic proteins is maintained. However, following radiation exposure as well as in the presence of various pathological conditions, a shift of this balance occurs due to changes in the expression of genes involved in apoptosis. In this respect, the study of the transcriptional activity of genes controlling cell proliferation and death is an important task of radiation biology as the disturbance of apoptosis promotes the development of pathological conditions accompanied by retention of cells with unlimited proliferative potential in the exposed body (Baryshnikov, Shishkin, 2002), or by the development of cytopenic conditions associated with increased cell death (Kvatcheva, 2000).

In studies conducted earlier in chronically exposed residents of the Techa riverside settlements, changes in the intensity of apoptotic death of the peripheral blood lymphocytes were registered in the long-term period (Blinova et al., 2020a). Moreover, changes in the transcriptional activity of apoptotic genes accompanied by a decrease in the relative mRNA content of the BCL-2 gene and increase in the relative content of mRNA of the BAX gene were demonstrated in exposed people 60 years after the onset of chronic radiation exposure (Nikiforov et al., 2020).

The next step of the work is the study of the relative content of mRNA of genes involved in cellular homeostasis in residents of the Techa riverside settlements, which are noted for the disturbed mechanism of cell elimination, in particular by increased intensity of apoptotic and necrotic cell death.

In this regard, the objective of the current study is to perform quantitative assessment of the content of mRNA of TP53, MDM2, ATM, BAX, BCL-2, CDKN1A, OGG1, XPC, PADI4, MAPK8, NF-KB1, STAT3 and GATA3 genes in the long-term period in chronically exposed residents of the Techa riverside villages who had an increased frequency of peripheral blood lymphocytes (PBL) at different stages of apoptosis and necrosis.

## Materials and methods

The study objects were the PBL of the 132 residents of the Techa riverside settlements who had been chronically exposed in 1949–1950 (Akleyev, 2016). Mean accumulated dose to the red bone marrow of all the exposed individuals was 426.4 ± 48.2 mGy (1.3–2930.0 mGy), mean accumulated dose to the thymus and organs of the peripheral immune system was 58.9 ± 7.9 mGy (0.1–489.0 mGy). Mean age of exposed people at the time of examination was 68 ± 0.6 years (55–86 years).

The control group consisted of 32 people who were not chronically exposed and lived in similar social and economic conditions. Mean age of the control group members at the time of examination was 67 ± 1.25 years (57–81 years). Intensity of apoptosis and necrosis was calculated in the control group according to the formula (1). For the purpose of the study the exposed people were subdivided into the following groups: exposed people with the intensity of apoptosis/necrosis exceeding the critical value, and exposed people with the intensity of apoptosis/necrosis within the normal range.

**Formula.**
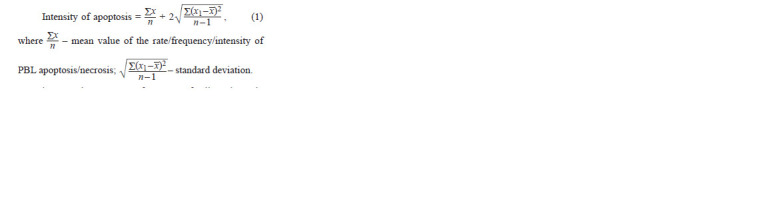
Formula

In the control group, mean frequency of cells at the early stage of apoptosis was 3.04, standard deviation – 4.52; at the late stage of apoptosis – 0.03, standard deviation – 0.06; at the stage of necrosis – 0.02, standard deviation – 0.04. Thus, the critical value of the frequency of cells for the early apoptosis was 12.08, for the late apoptosis – 0.15, for necrosis – 0.1. Exposed people who had frequency of cells at various stages of apoptotic death exceeding the critical value were included into the group with increased frequency of PBL apoptosis/necrosis. The description of the studied groups is given in Table 1.

**Table 1.**
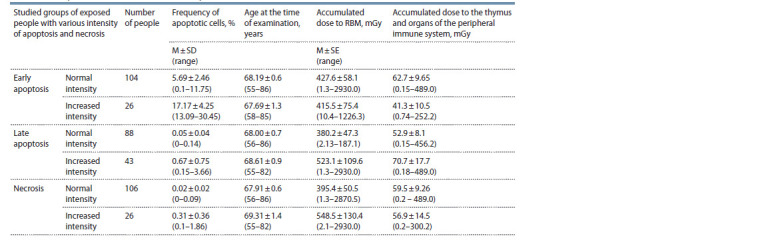
Description of the individuals under study

Blood for the study of PBL apoptotic/necrotic death was taken from the cubital vein in a volume of 6 ml into Vacuette tubes with Li-heparin (Improvacuter, China). The study was performed using flow cytometer with the Annexin V FITC stain kit (BD, France). Leukocyte fraction was isolated in Ficoll-Urografin density gradient from whole blood (density 1077–1078 g/cm3) in accordance with the standard method (Kheifets, Abalakin, 1973). Annexin-V (human) recombinant (FITC conjugate) and DNA binding fluorescent dye propidium iodide (PI) were added to cell-suspension. The analysis was performed using flow cytometer Navios (Beckman Coulter, USA). In the course of the analysis, cell populations at the stages of early and late apoptosis, and necrosis, as well as live cells were isolated. Results were given as a percentage ratio of cells that entered this or that stage of apoptosis and necrosis (see Table 1).

Blood for measuring mRNA relative content was taken from the cubital vein in a volume of 3 ml in sterile Tempus Blood RNA Collection Tubes (Applied Biosystem, USA). RNA was isolated through a column-based method with the Tempus Spin RNA Isolation Kit (Applied Biosystem). Information on concentration and purity of the isolated RNA samples was obtained using a NanoDrop 2000С spectrophotometer (Thermo Scientific, USA). Purity of the samples was measured by the values of absorption at wavelength of 260 and 280 nm (А260/280). The ratio of absorbances measured at A260/280 for purified RNA extracted from all blood samples was 2.1 ± 0.02. The total yield of the RNA was from 50 to 90 μg/ml. Reaction of reverse transcription for the cDNA synthesis was performed using the High-Capacity cDNA Reverse Transcription Kit (Applied Biosystem). Relative quantitative content of the mRNA was measured with RT-PCR using the CFX96 Touch amplifier (Bio-Rad Laboratories, USA)

The relative amount of mRNA in the studied samples was determined using 2–ΔΔСt-method. The data were evaluated with respect to the relative level of mRNA of the “housekeeping” COMT and B2 M genes and control group averaged values. Amplification curves were analyzed with the Bio-Rad CFX Manager 2.1 (Bio-Rad Laboratories) using the threshold line method. The calculation was performed taking into account three replicates for each gene and the efficiency of amplification was obtained by constructing calibration curves. Oligonucleotide sequence of the primers, temperature conditions of the RT-PCR were taken from international published papers and adapted to our experiments. The characteristics of primers are described in detail in (Blinova et al., 2020b; Nikiforov et al., 2020).

Statistical processing of the obtained data was performed using Statistica 10.0 and SigmaPlot software packages. The Kolmogorov–Smirnov test was used to check if the data in the samples were normally distributed. Since many of the investigated parameters did not have normal distribution, the non-parametric Mann–Whitney U-test and the Kruskall–Wallis test were used to compare the groups. The results were given as mean values, error of mean and range of the data (M, min–max) (Tables 2–4).

**Table 2.**
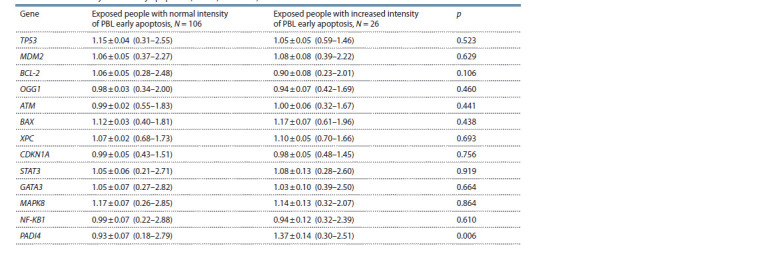
Relative content of mRNA (rel. un.) of the genes in the groups of examined people with different intensity of PBL early apoptosis (М ± SE; min–max)

**Table 3.**
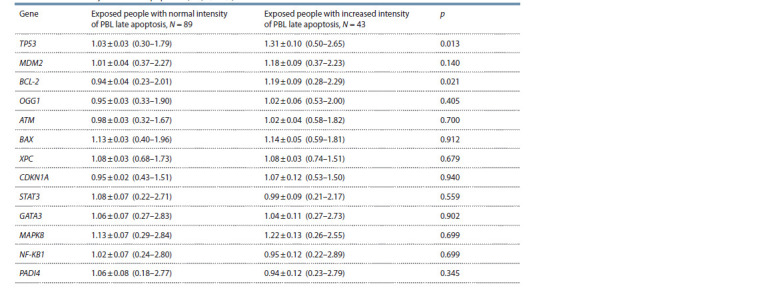
Relative mRNA content (rel. un.) of genes in the groups of examined people with different intensity of PBL late apoptosis (Ме; Q1–Q3)

**Table 4.**
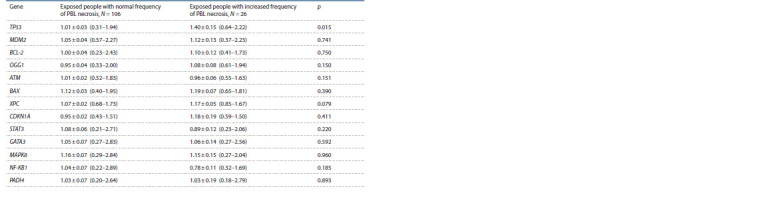
Relative mRNA content (rel. unit) of genes in groups of exposed people with various intensity of PBL necrosis (М ± SE; min–max)

Correlation-regression analysis performed without taking into account the outliers was used to reveal the dependences of changes in the relative mRNA content in the studied genes on radiation factors (dose to RBM, thymus and organs of the peripheral immune system). p-value ≤ 0.05 corrected for the multiple comparisons was used to exclude errors in the hypothesis wording.

## Results

Transcriptional activity of genes in chronically exposed people with increased intensity of early apoptosis

In the framework of the current study, a statistically significant increase (1.5 times) of the relative content of mRNA of the PADI4 gene was registered in the group of exposed people with increased intensity of the PBL apoptosis relative to the exposed people with normal intensity of early apoptosis (see Table 2).

**Fig. 1.**
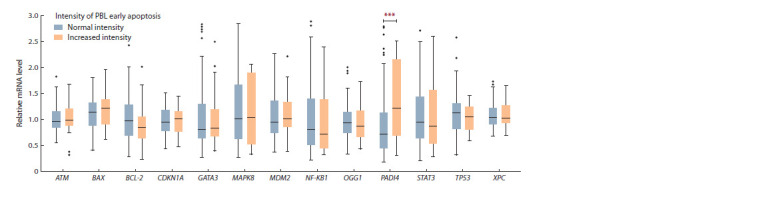
Distribution of the relative content of mRNA of the studied genes in chronically exposed people with normal and increased intensity of PBL early apoptosis. Here and in Figures 2 and 4 the data are presented as the median (25 and 75 percentile) and range (min–max); *** the differences between the groups are statistically signif icant (р < 0.05).

It can be seen from Fig. 1, that the changes in relative mRNA content of the PADI4 gene are due to the shifts of median data to the area/region of high values in the group of chronically exposed people with increased intensity of early apoptosis of the PBL, and not due to the changes in the transcriptional activity of this gene in some exposed people.

No statistically significant dependences were revealed when we checked the relationship between the mRNA content and dose characteristics (accumulated dose to RBM, thymus and organs of the peripheral immune system) in the group of exposed people with increased intensity of early apoptosis.

Transcriptional activity of genes in chronically exposed people with increased intensity of late apoptosis

**Fig. 2.**
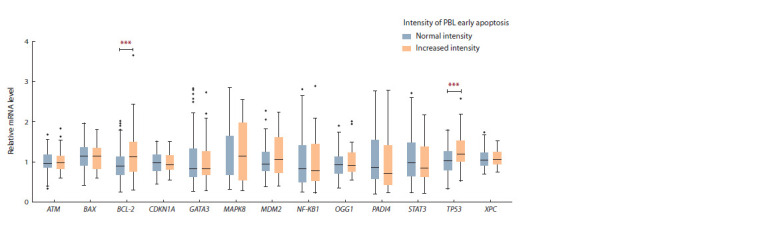
Distribution of the relative content of mRNA of the studied genes in chronically exposed people with normal and increased intensity of PBL late apoptosis.

In the study of the late stage of apoptosis, it was observed that chronically exposed people with increased intensity of late apoptosis have a statistically significant increase in the mRNA content of TP53 and BCL-2 genes relative to the exposed people with normal frequency of PBL at the late stage of apoptosis (see Table 3, Fig. 2).

**Fig. 3.**
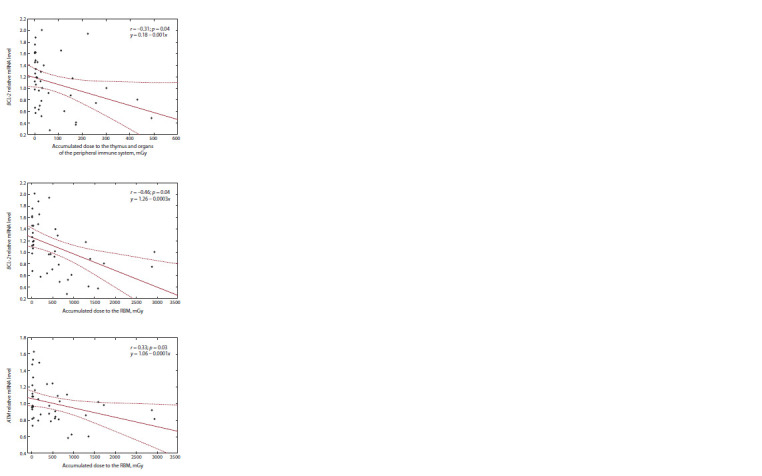
Linear dependence of the changes of the mRNA relative content of the ATM and BCL-2 genes on the accumulated dose to the RBM, thymus and organs of the peripheral immune system in the group of chronically exposed people with increased intensity of PBL late apoptosis.

As a result of the correlation analysis, negative correlation was revealed between relative content of the mRNA of the BCL-2 (r = –0.6; p = 0.001) and ATM (r = –0.4; p = 0.02) genes and dose to RBM in chronically exposed people with increased intensity of PBL late apoptosis. In addition, a negative correlation between the content of mRNA and dose to the thymus and organs of the peripheral immune system was noted for the BCL-2 (r = –0.4; p = 0.002) gene. The obtained dependences were studied using linear regression analysis (Fig. 3).

Transcriptional activity of genes in chronically exposed people with increased intensity of necrosis

**Fig. 4.**
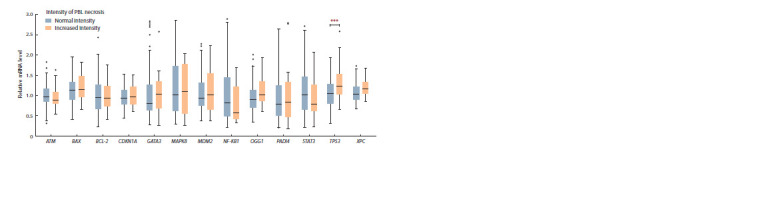
Distribution of relative mRNA content of studied genes in chronically exposed people with normal and increased intensity of PBL necrosis.

Statistically significant differences between the groups of exposed people with different intensity of PBL necrosis were shown only for the TP53 gene. An increase in the relative mRNA content of the TP53 gene (almost by 1.5 times) was observed in chronically exposed persons with increased PBL intensity of necrosis compared to chronically exposed individuals with a normal frequency of PBL that entered the phase of necrosis (see Table 4, Fig. 4).

**Fig. 5.**
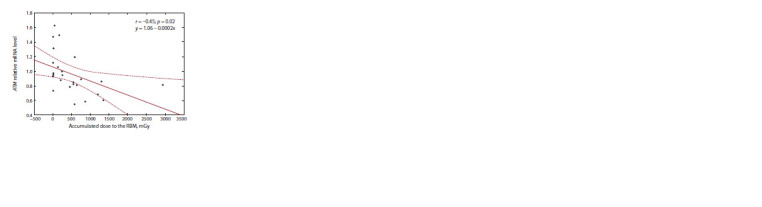
Linear dependence of changes of relative mRNA content of ATM genes on accumulated RBM dose in the group of chronically exposed people with increased intensity of PBL necrosis.

Negative correlation of the relative mRNA content of the BCL-2 (r = –0.47; p = 0.02) and ATM (r = –0.6; p = 0.001) genes with RBM dose was registered in the group of exposed people with increased PBL frequency at the stage of necrosis. The results of linear regression analysis showed no significant dependence of changes of mRNA amount of the BCL-2 gene on the accumulated RBM dose (p = 0.13), while a statistically significant negative linear dependence of mRNA content on RBM dose was shown for the ATM gene in the group of chronically exposed persons with increased intensity of PBL necrosis (Fig. 5).

Verification of the relationship between relative mRNA content and intensity of necrotic cell death revealed a negative correlation for the MAPK8 gene (r = –0.62; p = 0.01) in exposed people with increased frequency of PBL that entered the phase of necrosis

## Discussion

This study showed that chronically exposed people with increased frequency of PBL in the early stage of apoptosis have increased mRNA content of PADI4 gene compared to exposed people with normal intensity of early apoptosis. PADI4 is a Ca2+-dependent enzyme that catalyzes protein citrullination in the presence of Ca2+ (Rogers et al., 1977). In particular, PADI4 can mediate histone H3 citrullination on the promoters of p53 target genes such as CDKN1A, BAX, BCL-2, etc, and also bind to the p53 C-terminal regulatory domain, which causes repression of its activity (Tanikawa et al., 2012). In this regard, we can assume that PADI4 protein is an important mediator of the p53 signaling pathway that can lead to apoptosis activation.

In the group of exposed people with increased intensity of PBL apoptotic death at a late stage, modification of transcriptional activity of TP53 and BCL-2 genes is observed. In particular, a statistically significant increase in the relative mRNA content of these genes has been shown.

One of the main functions of p53 is induction of signaling mechanisms aimed at the elimination of potentially harmful cells (Miyashita et al., 1994). However, against the background of increased transcriptional activity of TP53, there is an increase in the anti-apoptotic BCL-2 gene in the group of exposed people with increased intensity of apoptosis. At the same time, the amount of mRNA of the BCL-2 gene decreases with the increase of dose to RBM, thymus and organs of the peripheral immune system. At this stage of work, it is difficult to explain this phenomenon; there is probably a violation of the mechanism of cell elimination against the background of hyperexpression of anti-apoptotic factors in some exposed people with increased intensity of apoptosis. This is also indicated by the fact that the transcriptional activity of the TP53 gene is increased in an exposed person with increased intensity of necrosis.

Moreover, negative correlation between the relative mRNA content of ATM genes and RBM dose was recorded in exposed people with increased intensity of late apoptosis. ATM gene dysfunction leads to a progression of genome instability, which is primarily accompanied by increased frequency of chromosome aberrations (telomere length shortening, increased level of paired and single chromosome fragments and frequency of translocations) (Hahn, Weinberg, 2002; Franco et al., 2006).

It is possible that down-regulation of this gene transcription, which increases with an increasing dose to red bone marrow of the residents of the Techa Riverside villages, is associated with depletion of intracellular reserves for neutralizing the resulting DNA damage, and thus is the leading cause of the increased intensity of cell death.

In the group of chronically exposed people with increased intensity of necrosis, a decrease in relative mRNA content of MAPK8 gene with increased intensity of PBL necrotic death is observed against the background of increased transcriptional activity of the TP53 gene.

MAPK8 phosphorylates hundreds of substrates responsible for stress response control and apoptosis regulation, including p53 (Guimaraes, Hainaut, 2002). In addition, MAPK8 phosphorylates BMF (BCL-2 modulating factor) on specific serine residues located inside and immediately adjacent to the BMF binding domain. BMF released from actin enters mitochondria, physically interacts with the BCL-2 protein, which subsequently also initiates apoptosis (Puthalakath et al., 2001).

## Conclusion

Thus, in the framework of the study it has been noted that changes in the transcriptional activity of apoptotic genes are primarily registered in exposed people with increased intensity of apoptosis, which is consistent with current ideas about the activation of programmed cell death. It was shown that gene expression depends on the stage of PBL apoptosis.

The study should be continued with an expanded sample of examined people and studied targets, which will allow defining the significance of parameters of transcriptional activity of some genes as markers of cancer and non-cancer incidence risk associated with apoptosis registered in chronically exposed people in the long-term period.

## Conflict of interest

The authors declare no conflict of interest.
